# Spaces for Citizen Involvement in Healthcare: An Ethnographic Study

**DOI:** 10.1177/0038038514544208

**Published:** 2015-06

**Authors:** Alicia Renedo, Cicely Marston

**Affiliations:** London School of Hygiene & Tropical Medicine, UK; London School of Hygiene & Tropical Medicine, UK

**Keywords:** acts of citizenship, citizenship, health, participation, participatory space, patient and public engagement, patient and public involvement

## Abstract

This ethnographic study examines how participatory spaces and citizenship are co-constituted in participatory healthcare improvement efforts. We propose a theoretical framework for participatory citizenship in which acts of citizenship in healthcare are understood in terms of the spaces they are in. Participatory spaces consist of material, temporal and social dimensions that constrain citizens’ actions. Participants draw on external resources to try to make participatory spaces more productive and collaborative, to connect and expand them. We identify three classes of tactics they use to do this: ‘plotting’, ‘transient combination’ and ‘interconnecting’. All tactics help participants assemble to a greater or lesser extent a less fragmented participatory landscape with more potential for positive impact on healthcare. Participants’ acts of citizenship both shape and are shaped by participatory spaces. To understand participatory citizenship, we should take spatiality into account, and track the ongoing spatial negotiations and productions through which people can improve healthcare.

## Introduction

Governments and development agencies worldwide encourage citizen participation in healthcare, with participation increasingly framed as a right of citizenship (Gaventa, 2002). In the UK, healthcare providers are required to involve patients and the public (e.g. Department of Health, 2007). Yet what is meant by public and patient participation, or engagement, or involvement, is often unclear and open to multiple interpretations and rationales (e.g. empowerment, democratic accountability, technocratic excellence), which in turn are linked to divergent ideas about the roles citizens could or should play in shaping healthcare (Martin, 2008; Tritter, 2009).

Policy and academic discussions of participation are permeated with spatial metaphors (e.g. ‘opening-up’, ‘widening’ ‘arenas’ and ‘spaces’ for public involvement, citizens gaining ‘positions’ of influence) (Cornwall, 2002: 2; Kesby, 2005), and with references to the importance of context in explaining successful or unsuccessful public and patient involvement. Yet spaces where participation happens and the ways spatial elements influence participants are rarely analysed. In this article, we examine how participatory spaces affect the participation that takes place within them, drawing on empirical examples from patient and public involvement initiatives in the UK.

We view participation in healthcare as part of a range of practices through which citizenship is negotiated and enacted. Citizenship is often mentioned in the participation literature, but rarely defined there. Here, we follow Isin, Gaventa, Barnes and others who argue that citizenship is more than a set of civil rights and responsibilities conferred on individuals in order that they participate in systems of state governance: it is a dynamic social practice with public participants actively negotiating with official bodies to determine how entitlements and duties are realised in practice (Barnes, 1999; Barnes et al., 2004; Gaventa, 2004; Isin, 2008). In this dynamic view of citizenship, what Isin (2008) has called ‘acts of citizenship’ become important, that is, deliberate actions through which citizens claim rights and common goods (e.g. quality healthcare) and through which they ‘constitute themselves (and others) as subjects of [those] rights’ and as actors ‘with “the right to claim rights”’ (Isin, 2009: 371). For an act to be an ‘act of citizenship’, it must help create new ‘scenes’ of action (Isin, 2008, 2009) and introduce ‘ruptures or beginnings’ (Isin, 2008: 27) in established ‘scripts’ and (spatial) orders. Acts of citizenship, therefore, are defined as requiring agency, as being necessarily transformative and promoting social change (Isin, 2008). For participation in healthcare to be a means of enacting citizenship, then, participants must act as ‘makers and shapers’ not simply ‘users and choosers’ (Cornwall and Gaventa, 2000) of health services.

Conceptualizing citizenship as a dynamic social practice requires us to attend to how citizenship is negotiated in time and space. We know that citizenship cannot be conceptualised ‘aspatially’ (Massey, 1995: 284), yet the ways spatial aspects of participation mediate how citizenship is realised in practice remain unexplored. In this article, we examine specific participatory spaces and show how public participants perform acts of citizenship that help influence healthcare improvement. We develop a framework for understanding the link between spatiality and citizenship.

## Spatiality and Participatory Citizenship

We draw on Massey’s conceptualization of space as ‘ongoing constructions’ (Massey, 2005: 180), in a constant process of becoming, through three mutually constituted and constituting dimensions (Massey, 1992, 2005): the physical, the temporal and the social.

Space is physical: it is constructed and localised in places and crystallised in practices, which have material consequences simultaneously framing interactions between people, shaping their embodied experiences and social processes and being constituted by them. Space is both the ‘medium’ constraining social relations and social structure and an ‘outcome’ or manifestation of those social relations and social structure (Soja, 1989: 129).

All of these interactions occur over time as well as physical space and because material and social aspects also shape how future events play out, they become constitutive forces of the temporal (Massey, 1992, 2005).

Space is social, comprising networks of social interactions, trajectories (from local to global) and practices (Massey, 1999, 2005), and permeated by difference, power and resistance (Massey, 1992; Allen, 1999). Access to and mobility through space is negotiated, contested and imbued with struggles between different groups over their diverse uses of, perceptions about, and aspirations for space (Aldred and Jungnickel, 2012), while the material dimension of space frames socio-cultural and political processes (Harvey, 1990; Lefebvre, 1991; Soja, 1989).

Analyses of participation often focus on social dimensions (Cornwall, 2002; Kesby, 2007), particularly unequal power relations between professionals and participants (Cornish, 2006). For instance, bureaucratic interactions between professionals and participants that govern ‘invited’ participatory spaces (Cornwall, 2002) (that is, officialised spaces created and managed by professionals and statutory officials) can undermine participants’ agency (Cleaver, 1999; Cleaver et al., 2001) and the development of truly collaborative partnerships (Martin and Finn, 2011; Stern and Green, 2008). Professionals’ bureaucratic priorities also affect the temporal characteristics of invited spaces (e.g. durability and rhythm of participatory processes) often neglecting participants’ own circumstances and opinions about the time needed to achieve change (Ruiz, 2004). Participation is also affected by the public participant identities that citizens develop through their interactions with health professionals (Renedo and Marston, 2011).

Far less attention has been paid to the material dimensions of participation (Kesby, 2005). Cornwall (2008) notes that participatory settings have a history and consequently symbolic connotations for participants that shape social dynamics within them affecting not only who participates but also what is ‘sayable’ and ‘doable’ within participatory spaces (e.g. whether people are suspicious of participatory initiatives or lack confidence to enter participatory locations) (Cornwall, 2002). Participants’ voices and forms of knowledge can also be constrained and shaped by material practices such as formal tools and techniques professionals use to manage participatory processes in invited spaces (Jupp, 2008; Kothari, 2001).

All these spatial features help explain how formal invitation into participatory spaces is not enough for individuals to act with agency to influence and claim their rights to quality healthcare. We know that even with power imbalances and spatial constraints, citizens can potentially exercise agency (Cornwall, 2002; O’Toole and Gale, 2008), and find possibilities for enacting citizenship. The ways this is mediated by spatial dimensions of participation, however, has not been investigated empirically. Following O’Toole and Gale (2008), we draw on De Certeau’s (1984) distinction between the interrelated concepts of ‘strategies’ and ‘tactics’ to understand the dialectic between structure and agency, that is, between governance structures that produce and manage temporal, social and physical aspects of invited spaces and participants’ agency to re-shape these spaces.

The spatial is simultaneously a sphere of constraints and opportunities where ‘there are always connections yet to be made’ and interconnections that ‘may or may not be accomplished’ (Massey, 2005: 11). Thus the spatial allows for the possibility of agency and action (De Certeau, 1984). This relational and open characterisation of space has been criticised for being too abstract and metaphorical (e.g. Malpas, 2012). Yet Massey’s relational conceptualisation helps us examine the potential for people to engage in acts of citizenship that alter the spaces that shape their living and that are governed by more powerful actors, to pursue their own interests and projects. This is certainly relevant in the context of invited spaces, which while institutionally framed and controlled by their creators (Cornwall and Coelho, 2006) nevertheless ostensibly require citizens to influence healthcare. We see participatory space as constructed and reconstructed through ‘strategies’ and ‘tactics’, allowing involvees to have ‘spatial agency’ (Sewell, 2001: 54), using invited spaces for their own ends whilst producing an effect on them.

We explore here how participants manipulate the multiple dimensions of ‘invited’ participatory spaces (Cornwall, 2002) to perform acts of citizenship. We conceptualise participatory citizenship as a process of engaging with, negotiating and re-constructing space. We argue that acts of citizenship in healthcare must be understood in terms of the spaces which shape them, and which they shape in turn.

## Methods

This research is part of a larger ethnographic project exploring the patient involvement activities of the Collaboration for Leadership in Applied Health Research and Care (CLAHRC) for North West London. CLAHRC helps the UK National Health Service (NHS) implement evidence-based research to improve quality of patient care. We obtained approval for the study from both NHS and London School of Hygiene and Tropical Medicine Research Ethics Committees.

We draw on 22 in-depth 60–120-minute individual interviews (10 women, 12 men) and intensive ethnographic work including planned observation (132 hours) of official patient and public involvement activities from 2009 to 2013. These included: monthly meetings at hospitals of healthcare professionals and patients working together on healthcare improvement projects (e.g. to try to improve particular health services); steering groups where patients helped organise CLAHRC conferences, were consulted about commissioning CLAHRC improvement projects, or discussed CLAHRC programme strategy (all conducted at hospital meeting rooms or (rarely) at a third sector organisation office); and training for involvees – service users or members of the public involved in CLAHRC activities – to help them become ‘effective’ patient representatives (CLAHRC-funded and held at a third sector organisation office). Alicia Renedo (AR) conducted additional opportunistic observation regularly over the four-year project at other CLAHRC events where patient involvement was not the main aim but where patients (including some interviewees) were present. These included CLAHRC conferences to support healthcare teams’ learning about improvement methods (including patient involvement, conducted at various healthcare professional associations, e.g. Royal College of Physicians), and meetings to discuss the developments or future funding of the CLAHRC programme (all meetings held in hospital meeting rooms). The ethnographic fieldwork gave AR an experientially-rooted insight into the nature of participatory spaces, and a longitudinal perspective on the evolution of processes and relationships within these spaces. It also offered multiple opportunities to keep abreast of progress of participatory activities. Interviews were with CLAHRC involvees and covered experiences of participating in healthcare improvement, focusing on contextual information (what, when, where, with whom). Interviewees gave accounts of their participatory pathway into CLAHRC, e.g. their experiences, motivations to get involved, and factors affecting their participation over time. This article reflects interviewees’ participation in diverse invited spaces, not only with CLAHRC. To preserve anonymity, we omit or alter identifying participant details here, including details about their projects.

Through observation of patient involvement activities we examined contextual aspects of participation and how involvees used participatory spaces when interacting with one another and with healthcare professionals. Following Emerson et al. (1995), we focused on processes and practical aspects of participation: what actually happens when people participate, and what practices they develop to navigate the conditions and constraints they encounter.

We analysed interviews and field notes using iterative thematic analysis (Attride-Stirling, 2001) to identify key themes for each of the three interwoven dimensions of participatory space. Our coding frame reflected our a priori interest in these spatial dimensions, and was also developed inductively from the entire data set. The frame helped categorise data in terms of the physical places and temporality of participation, as well as the social processes, interactions and trajectories through which participation developed over time and physical space (Massey, 1992, 2005). For instance, codes included ‘moving across’, ‘connecting’, ‘learning institutional workings’, and ‘negotiating access’, which spoke about the trajectories and practices participants developed to manoeuvre within the constraints of given participatory spaces (De Certeau, 1984; Massey, 2005). During repeated rounds of coding, re-coding, and ‘memo-writing’ (Charmaz, 2006), we made frequent comparisons across the interview and field note data to generate, review and refine themes (Braun and Clarke, 2006), complementing this with narrative analysis of each involvee’s interview and observed actions (Riessman, 1993). The narrative analysis illuminated how involvees’ participatory experiences unfolded through time and across different settings and sequences of events, making links between involvees’ participatory actions and consequences (How did involvees’ actions develop through interactions with healthcare professionals in particular settings and at different times?). Three tentative conceptual categories emerged from the analysis (‘mapping’, ‘cross-pollinating’ and ‘bridging’), which described the tactics involvees developed to influence healthcare within participatory spaces. We continued to question and refine these initial categories to delineate their properties and their interrelationships. At this point, we integrated citizenship theory into the analysis, went back to the data and written memos and compared the emerging categories with the concept of acts of citizenship (Isin, 2008). We questioned the relevance of each category for explaining whether and how participants’ attempts to influence healthcare helped create new ‘scenes’ of action to help influence healthcare by bringing ‘beginnings’ (Isin, 2008: 27) and ‘openings’ (Massey, 2005) to invited participatory spaces. This iterative and reflexive process prompted further refinement of categories into ‘plotting’, ‘temporary combination’ and ‘interconnecting spaces’ – categories which captured better what participants were trying to do within the available spaces they found themselves in to influence and claim right to quality healthcare.

Interviewees were typical of the participants we saw more generally in that almost all were white, educated, and had professional backgrounds. Interviewees were retired (13 interviewees), unemployed (2 interviewees), or were not involved in paid employment because of chronic illness or having become carers (5 interviewees). Their mean age was 65 years (also typical). There was little evidence of involvees coming from ‘hard-to-reach’ groups, although in two cases, interviewees’ first language was not English.

Interviewees and other patient/carer involvees we met during fieldwork were participants in many settings (at the local and national level, inside and outside invited spaces). For instance, in healthcare via transient (e.g. consultations about commissioning clinical research, advisory meetings about CLAHRC strategy) and more durable formations (e.g. patient groups, governance boards, service improvement projects, research ethics committees); in health and social care via voluntary patient organisations, and Local Involvement Networks (state-created structures for public involvement hosted by third sector organisations).

## Creating Productive Spaces

Despite the limitations of the existing invited participatory spaces, described below, our observations and interviews revealed how involvees were able to reconfigure some spaces. We classify their actions into three conceptual categories: ‘plotting’, ‘temporary combination’ and ‘interconnecting spaces’.

All participants belonged to particular patient/carer communities and talked about sharing specific goals from those communities, typically about improving the quality of specific health services for that community (e.g. integrated care pathways for diabetic patients, quality and compassionate care for elderly people, specialist care for a rare genetic disorder). We coin the term ‘loyalty projects’ here to denote participant orientation towards community goals and to distinguish such activity from more individualistic projects.

### Plotting

Our observations and interview narratives about accessing and moving across invited spaces suggest involvees simultaneously positioned themselves as guests and fighters struggling to have an effect and pursue their loyalty projects. They used spatial vocabulary to articulate their participatory experiences (‘overcoming inertia’, ‘being in the way [of professionals]’, ‘meandering’) and described their practices as ‘chugging’ or ‘lumbering’. These experiences were framed by professionals’ control over the production of material, temporal and social aspects of invited spaces.

Being a ‘guest’ was inevitable when bounded participatory locations were chosen by health professionals and statutory officials and situated in places ‘owned’ by them – institutional settings with restricted access, away from involvees’ homes and neighbourhoods, (e.g. town hall meeting rooms, hospital boardrooms). Involvees lacked control over physical access to these spaces; for instance, they had to undergo CRB checks and training to become ‘official’ (patient) representatives, and required material artefacts such as invitations or institutional electronic cards to enter meeting rooms (Quote 1).

You had to fill in a form, and you had to be CRB checked [to be part of a healthcare services quality inspection team]. [. . .] And when we go and do a visit this badge is given to us to wear at all times [at the hospital]. But we’re not allowed to keep it just in case we go off and do something off our own bat I suppose. (Quote 1, Participant A. Quotes are from interviews unless otherwise stated)

As ‘guests’ involvees had to accrue detailed knowledge about social, material and temporal aspects of invited spaces (e.g. workings of the NHS, professional hierarchies, power dynamics, bureaucratic procedures, institutional decision-making time frames), which they wished to change to benefit their loyalty projects. They used the knowledge to try to mould relationships with involvers, or speed of decision-making so that they could turn professionals’ influence to their own advantage (Quote 4). We have termed this group of tactics ‘plotting’ – a term used by one of our interviewees (Quote 2) which captures both the ‘mapping out’ and ‘scheming’ aspects of these processes. Participants ‘plotted’ in order to navigate within and across invited spaces and ultimately to pursue their loyalty projects (Quote 2). ‘Plotting’ was not only about mapping out invited spaces but also creating new ‘scenes’ (Isin, 2008) of action by forging a socio-temporal and physical ‘pathway’ to allow the participant ‘to meander in a position of influence’ (Quote 2) within them.

I developed very quickly a detailed understanding of the [healthcare] system [. . .] I had to find out everything myself [. . .] you have to sort of fight to create your own space so that you can operate and we’ve spent a lot of time in the last year forcing our way in [. . .] you had to do an awful lot of research and background checking as to what the processes were that were available to you [. . .] how you actually used the processes available to your advantage to beat the system [. . .] when you’re sitting in a cabinet meeting at the council or something of that level, you can ask four or five questions and steer an agenda in a very specific way [. . .] the system is designed and set up in such a way that you need those skills to plot your way through it. (Quote 2, Participant K)

This quote exemplifies the range of practices and degree of reflection participant K invested in plotting to create new scenes of action to influence healthcare improvement for elderly people; an explicit loyalty project for him in his multiple participatory experiences in healthcare services committees and commissioning boards since he had become an elderly person’s carer. Plotting was a way to pursue the right to quality healthcare within the confines of what was ‘doable’ and ‘sayable’ in those spaces; it was an attempt to perform acts of citizenship where acts were often circumscribed by established scripts of acceptable behaviour. Self-regulation was a core theme; interviewees spoke about and we observed them acting in a way that fitted what they perceived to be acceptable, such as conforming to institutional conventions (e.g. normative ways of intervening at meetings, keeping to the agenda) and interpersonal codes of conduct (e.g. assertiveness, wearing a suit) (Quote 3). These social factors shaped how participants could use and alter the space to plot and ‘force’ (Quote 2) their way in and were also manifest in the participants themselves as they developed new skills and new ways of being to adjust to these spaces.

Whatever I say rarely gets minuted [. . .] I don’t tend to ask many questions [at Diabetes Board meetings and health services committees where he represents diabetic patients like himself], mainly because there’s another user representative on there, who is a lot more erudite than I am. [. . .] He’s much better at it [questioning healthcare professionals]. [. . .] The point [is] saying it and knowing how to say it. [ . . .] if I can find some way of getting as good as my colleague that would be a start in getting the attention of the meeting. (Quote 3, Participant I)

Limited by proceedings that restricted how he could intervene at meetings, participant I learned it was more efficient to ‘butt in’ and address comments to specific Board members rather than to the group as a whole.

Involvees learned about the material practices needed to navigate the web of institutions that form the healthcare infrastructure (e.g. strategic use of emails and meeting minutes as documentary evidence of their requests to involvers). Some slipped between bureaucratic and colloquial language use (e.g. Quote 2 ‘processes available [. . .] beat the system’) – a linguistic manifestation of their navigation through unfamiliar territory. ‘Plotting’ not only enabled participants to move across invited spaces (e.g. learning how and who to lobby for changes in services) but also to bring ‘beginnings’ (Isin, 2008) to these spaces and alter the institutional practices and social processes that configured these spaces. ‘Plotting’ involved some involvees shifting established patterns and tactically navigating, stretching and blurring ‘official’ boundaries of invited spaces to become agentic within these spaces and work to achieve their own goals (Quote 2, Quote 4).

I said [to the ‘Overview and Scrutiny’ meeting Chair], ‘I’m bringing this up to ‘Overview and Scrutiny’ and before the meeting actually started the Chair came down and said ‘I’ve dealt with that. You don’t have to bring it up at the meeting now’. [. . .] It will be as an item on there that this has been dealt with out of the session, which was good. [. . .] If you go to a full-blown meeting and ask at a meeting, there’s so much inter-political arguing amongst themselves that you don’t really get a decision [. . .] You really need to catch them before the meeting starts or when you knock off halfway through for a toilet break. And say [. . .] ‘Can you make sure that this happens?’ (Quote 4, Participant L)

Participant L (Quote 4) tried to alter established NHS and local council institutional procedures and power relationships to pursue an alternative time frame (faster) to turn the possibilities of invited spaces into reality (that is, improving services for patients with the chronic condition he shared, and who he also represented as Vice-chair of a patient charity). He strategically presented himself at meetings with his Vice-chair title rather than as a patient representative and creatively used both informal spaces (‘toilet break’) and formal meeting procedures to make requests to professionals in relation to his loyalty project (commissioning a specialist service for his patient community). As with participants K and L (Quote 2, Quote 4), most involvees acted according to rules of behaviour defined by involvers and learned how to manoeuvre within them. ‘Plotting’ was the first step in shaping a participatory space of one’s own within invited arenas to relate more effectively to involvers (e.g. improved negotiation), to further loyalty projects, and to produce an effect in those arenas.

Participant L (Quote 4) frequently mentioned ways he had found to fight for quality services for his patient community by identifying and adapting to their norms and processes, bypassing the official rules of invited spaces. Cumulative experiences in participatory spaces helped involvees to acknowledge and renegotiate differences with involvers, and to rework invited spaces by producing ‘beginnings’ (Isin, 2008) to pursue their interests. The more the involvees were able to navigate the structures of invited spaces, the more they were invited or could initiate participation themselves, enabling them to expand their participatory space.

### Transient Combination

Participatory space was relationally constituted from social and material transactions involving participants doing things ‘for’ – rather than with – professionals. Within these social interactions there was a tendency towards one-way flows of information (involvees’ and patient community opinions solicited by professionals), of physical movement (involvees sent to professionals’ institutions rather than professionals going to involvees’ home turf) and of temporal resources (involvees’ time committed to professionals’ requirements). The temporality of each participatory formation (timing, sequencing, and frequency of activities) was controlled by involvers to fit their work schedules. Professionals brought involvees into their institutions, provided involvement opportunities, solicited their opinions, and referred them to other professionals tasked with ‘patient involvement’. Involvees were ‘traded’ between professionals keen to recruit participants to fulfil commissioners’ public involvement requirements.

Because invited spaces were physically fixed within institutions (hospitals, town halls), involvees were obliged to move around in order to participate. They moved across different participatory moments and places, from their homes, neighbourhoods and local community groups (e.g. residents’ associations) to third sector organisations, across invited spaces in healthcare institutions. An overarching theme of fragmentation emerged from participants’ accounts of mobility, with involvees speaking about trying to channel their involvement efforts in disparate participatory spaces onto one loyalty project, to ‘focus’ their multiple participatory engagements to make them more coherent and less fragmented. ‘Transient combination’ of elements of projects was a step towards this, a functional response to the polycontextual and disconnected nature of invited spaces. Yet as indicated by the name, participants could only make transient and sparse connections between invited spaces.

Involvees pooled material and social resources (funding, knowledge, relationships) from the disparate invited spaces, adding each participatory experience into their main loyalty projects through time and across different settings. This tactic helped them co-opt elements of invited spaces so they performed new functions. For instance, one participant used his participation in a national healthcare commissioning group and in a parliamentary group to raise ‘the profile’ of a local hospital project where he was helping redesign and improve services for patients who shared his chronic condition – improvements which were also a key concern of the national patient organisation (for the same condition) on whose board he served.

Combining new resources, even in a transient way, helped create a sense of control in their participatory space and a personal raison d’être within it (that is, the feeling that one was working towards achieving one’s loyalty project despite having to participate in spaces and projects owned and pre-established by professionals). Involvees often said or behaved as though they wished to use their participatory experiences to achieve additional goals, and this seemed to transform the meaning they attached to invited spaces (Quote 5). One participant combined involvement in co-delivering a conference with healthcare professionals into a loyalty project temporarily by using the conference space to publicise the project (she circulated leaflets about a voluntary organisation she also participated in which advocated for the welfare of patients like herself). Another participant (Quote 5) told us how he gathered feedback on and promoted implementation of a patient self-care management leaflet he had developed with his patient group for patients with his chronic condition while working on a nominally separate service improvement project.

[During training received as part of the improvement project] I was busy networking with the two people who delivered the course as a channel to pass on ideas that I’ve got elsewhere [self-care management leaflet ] [. . .] I’m extremely keen to spread [the leaflet] just as widely as I possibly can. [. . .] [After the course] I sent [the teacher] an email, with the [leaflet] [. . .] and asked her to share it with anybody else she thought would be interested. (Quote 5, Participant G)

Participant G also used his invitation to do a presentation (at the Town Hall) about the improvement project to promote and distribute copies of the self-care management leaflet. ‘Transient combination’ was implicitly linked to a desire to extend one’s impact beyond invited spaces. It was a way to take more control over those spaces and render them more productive. Involvees still had to respond to the requests of professionals and adjust to the frameworks of invited spaces but could temporarily make elements of invited spaces their own to use elsewhere.

Involvees recognised the value of occupying many invited spaces as a way of ‘being aware [of what is going on in healthcare services]’ or ‘collecting information’ that they could pass on to their patient communities. Moving into and out of disconnected participatory experiences (e.g. sitting on different committees) was reconstructed as a space of possibilities and used pragmatically to obtain resources.

‘Transient combination’ represents a step further than plotting: more than simply functioning in multiple existing spaces, it involves also using and transposing elements across them in an attempt to shape them. While ‘plotting’ is about finding a route to influence within a particular participatory space, ‘transient combination’ involves crafting a personal sense of place within one’s plural and mobile experiences in a participatory landscape. With this tactic, invited spaces were temporarily ‘inserted’ into involvees’ networks of associations and participatory experiences. There was a sense among involvees that they could always find something within the bounds and jurisdiction of invited spaces that could be transferred elsewhere to benefit their wider loyalty projects. For instance, one participant with disability accepted invitations to participate both on a service user group at the town hall and on a quality accounts consultation group at the hospital because they would enable her to gather information that she could report back both to the disability charity where she was a trustee and to her residents’ association (disability was common among the residents).

Crucially, these transient acts of citizenship only occurred during fleeting agentic moments in which participants shaped small stretches of participatory space.

### Interconnecting Spaces

Once inside bounded invited spaces, involvees became embedded within them: particular individuals became known to more and more professionals and invited to become involved in more and more tasks.

These highly-involved involvees participated frequently in various settings and increasingly embodied a web of personal connections and participatory experiences that they used tactically to turn invited spaces into landscapes of possibility. They used their plural occupancy, mobility and relationships within these settings to facilitate connections across invited spaces and enlarge their scope. That is, they linked people, healthcare improvement projects and processes from different participatory spaces and tried to overcome the limited temporal and spatial scope of these spaces. For instance, Participant L told us about integrating three separate healthcare organisations’ service improvement projects together, taking ideas and information from one to the other, and looking for commonalities between them. Participants functioned as hubs for new relationships and social processes (e.g. collaboration) within and across their local community, voluntary organisations and invited spaces in healthcare institutions. Involvees brought unconnected healthcare professionals from the same or different institutions into relationships with one another, so that they could improve healthcare together. This ability to link and catalyse was sometimes recognised by involvers – participant L told us about when one project lead had invited him to help with the project:

[The lead] said ‘because you know a lot of people, you know a lot of what goes on [. . .] you will be an ideal person to be able to push things along for us.’ (Quote 6, Participant L)

‘Interconnecting’ tactics were used to try to overcome the social and physical distance between invited spaces and to accelerate the evolution of these spaces (that is, alter the temporal organisation of spaces). These tactics reflected participants’ aspirations for their projects: to increase speed of implementation, to ensure sustainability over time, and to spread them to other healthcare organisations. As such, ‘interconnecting’ involved altering the scale of participatory spaces. Participant L (Quote 6) attempted to speed up improvements in services for people with a chronic condition like himself and expand them to other settings by creating links between diverse participatory spaces (different healthcare organisations’ service improvement teams and the national patient charity on whose board he served), synchronizing actions between them (e.g. design of new ‘care pathways’) and incorporating elements from one into another (e.g. patient leaflets designed by the charity). As well as interconnecting between healthcare professionals from different invited spaces, he also tried to raise patient awareness in community and patient groups about the intervention he helped develop hoping to help it continue beyond its initial funding period by increasing demand. Participant K (Quote 2) captures involvees’ concern with the temporal dimensions of participatory spaces (‘it can actually take quite a long time to make even a small change’). Through his interconnecting actions he tried to create new social processes to integrate disparate improvement efforts. At meetings, we often observed him offering ‘interconnecting’ help to professionals, for example by helping them pilot a patient medication management tool, putting them in contact with social care institutions, and linking to other NHS committees where he participated.

Involvees’ efforts to shape the future of improvement projects were often undermined by social processes (e.g. lack of control over institutional bureaucracies and over official timings) (Quote 7, Quote 8). Institutional bureaucratic controls (e.g. ‘clinical governance’) shaped the development of healthcare improvement projects and their sustainability, and as such were important constitutive forces of the temporality of participatory spaces and of participants’ use and experience of these spaces. Bureaucratic elements not only framed the temporal organisation of projects (evolution of activities) but also shaped material aspects of participatory spaces. Participants struggled to obtain resources (institutional approval, money) quickly enough to produce materials they needed for awareness-raising activities in their patient communities (Quote 8). Participant G’s and H’s (Quote 7, Quote 8) accounts capture the dynamic relationship between temporal and social dimensions of participatory spaces.

They [healthcare institution] were absolutely determined to do everything that they possibly could to put obstacles in the way [of involvees recruiting for and then delivering a training course for patients]. [. . .] The whole session [healthcare improvement project team meeting], which went on and on and on, was taken up with back to clinical governance [discussions about whether the project required ethics approval] [. . .] We still don’t have a final agreed governance rules for the project. They keep on changing. [. . .] These things tend to get stuck. (Quote 7, Participant G)

[Healthcare institution] has the copyright [of patient self-management tool] [. . .] [institution] has masterminded the official public launch [of the tool]. I would like to go on and do some work on that [i.e., raise public awareness] straight away but we, the patients have got to wait for [them to launch it] [ . . .] I want every GP and every nurse [on board]. I’m going to make sure everyone’s got [one]. (Quote 8, Participant H)

Through ‘interconnecting’ participants extended temporal and social boundaries. Participant H (Quote 8) used her participation in other invited spaces (e.g. primary care organisations) to promote the patient self-management tool she helped design at her local hospital for service users like herself, ensure its distribution, and monitor its use.

‘Interconnecting’ involved a more strategic and sustained form of agency than the ‘transient combination’ tactics. It was more evident among participants who had spent longer in invited spaces and who were more able to act creatively within the constraints of these spaces. While ‘transient combination’ tactics enabled participants to invert the one-way direction of flows of resources (that is, from involvees to professionals) on an ad-hoc basis, ‘interconnecting’ tactics had more permanent transformative impacts on invited spaces, actively creating new and multi-directional processes across spaces and building constellations of connections for healthcare improvement. ‘Interconnecting’ tactics assembled together fragments of the participatory landscape, addressing issues such as the lack of interaction between involvers, who were often unaware of each other and each others’ projects even when they addressed similar problems. Some healthcare professionals were aware of and in cases tried to harness the power of some involvees to move across extended networks of participation and occupy places and establish relationships with people where the professionals did not (e.g. parliamentary groups, patient groups) (Quote 6). ‘Interconnecting’ then, involved acts of citizenship that did not just bring ‘beginnings’ (Isin, 2008), but also more lasting change.

## Discussion

In this article, we have developed a framework for understanding participatory citizenship in healthcare. We argue that participatory spaces and citizenship are co-constituted. Participants’ acts of citizenship are both shaped by, and simultaneously shape participatory spaces, potentially making them more collaborative and productive. Citizens’ impact can also have broader spatial scope through acts of citizenship that traverse invited participatory spaces, but not everyone is able to transcend the spatial barriers of participation and enact citizenship.

Our framework helps structure analysis of what citizen participation means in practice: participatory spaces constrained participants who through their acts were nevertheless focal points in a complex participatory landscape. Participants began to shape the ways participation was implemented, and sometimes developed alternative and more agentic ways to enact citizenship to the ones formally offered within these spaces. Their acts of citizenship introduced ‘ruptures’ (Isin, 2009) in participatory spaces and created new ‘scenes’ (Isin, 2009) in which they could exercise their right to participate in shaping healthcare provision and demand collective entitlement to quality healthcare. Participants used different tactics to reconfigure spaces to try to increase impact on healthcare. Thus, participatory spaces were simultaneously the ‘outcome’ and constraining ‘medium’ (Soja, 1989) of participants’ acts of citizenship. Even while participants tactically articulated (or ‘earned’) their citizenship ‘from below’ in ways that created new opportunities to influence healthcare, they did not question the top-down constraints imposed on their participation.

Three conceptual classes of tactics emerged from our analysis – ‘plotting’, ‘transient combination’ and ‘interconnecting’ – each involving different degrees of citizenship agency in reworking participatory space. Involvees’ agency to function ‘tactically’ (De Certeau, 1984) to shape healthcare was framed by the boundaries created by the material, temporal and social practices, or governing ‘strategies’ (De Certeau, 1984) of those who manage and organise invited spaces (Cornwall, 2002, 2003) (e.g. their institutional settings, formal processes). This was particularly clear in the ‘plotting’ and ‘transient combination’ tactics, which involved acts of citizenship that brought ‘beginnings’ (Isin, 2008) and ‘openings’ (Massey, 2005) to invited participatory space but where agency to transform space was constrained by the nature of space itself. By drawing on resources and relationships in their web of participatory experiences, and shifting them over time and across physical space, involvees also performed ‘interconnecting’ acts of citizenship that interrelated and expanded participatory spaces, creating new circumstances for influencing healthcare. The spatial assemblages that they constructed through these ‘unofficial’ interconnecting acts of citizenship still involved struggle, but were less fragmented than the original invited spaces, involving more interdependencies between healthcare professionals, patients and civil society. ‘Interconnecting’ and ‘transient combining’ acts of citizenship can potentially make participatory landscapes more collaborative and extensive in scope, and engage the networks of people and actions needed for sustained healthcare improvements. Participatory spaces can be expanded and become ‘topologically’ different through acts of citizenship that interconnect discrete processes within these spaces to stretch spatial boundaries, and make healthcare improvement a more collective effort. Acts of citizenship can thus contribute to altering the scale of invited participatory spaces. Our work, then, shows what it means to say that space is made and remade through relations and interconnections at all levels (material, social and temporal) (Massey, 2005), and how one way this happens in practice is through acts of citizenship. Acts of citizenship are integral to the realisation of the ‘possibilities’ (Massey, 2005) that participatory space offers.

Citizens may thus influence healthcare in ways not obvious to the people inviting them to participate; they may act creatively ‘outside’ the ‘already-scripted’ forms of citizenship (Isin, 2009) and the ‘framing mechanisms’ (Craig and Porter, 1997) that manage their actions in the invited spaces. Individuals can have broader (spatial) impact through acts of citizenship that traverse participatory practices located ‘around the table’ (Stern and Green, 2008) of meetings and committees.

Involvees in our study were seasoned participants, and it is not clear whether others without previous participatory experiences would have had similar access to and mobility across participatory spaces. Interconnecting tactics in particular were possible because of individuals’ multiple spatial trajectories, which they drew on to give the participatory landscape alternative topologies. They occupied many invited spaces, were mobile across them, and had access to other third sector and community spaces. These diverse experiences and spatial trajectories may explain how they were able to exercise agency to coordinate actions, engage stakeholders and mobilise resources across spaces, creating new temporal, social and material interconnections across them. Participants’ involvement in diverse invited spaces can potentially increase the technical knowledge required for effective participation (Cornwall, 2002), as we have seen with our participants’ ‘plotting’ tactics. ‘Professionalization’ of participants is a well-documented phenomenon (El Enany et al., 2013) and our study illuminates how this happens in (spatial) practice. Involvees we encountered were highly knowledgeable about health and social care policies and institutions. Healthcare professionals may actively educate and invite such ‘professional’ involvees, potentially excluding others, and making patient and public involvement initiatives less likely to include certain groups (El Enany et al., 2013).

We have shown how participatory spaces and citizenship are co-constituted and offer a way to conceptualise the spatiality of participation that helps understand the complexities of enacting citizenship in healthcare which are likely to be relevant outside our specific study location – both elsewhere in the UK and more widely. Citizenship in participation is an ongoing spatial negotiation and production enacted through networks of socio-temporal and material practices and trajectories. Participatory citizenship is clearly not determined solely by individual skills or official structures for public involvement. The agency of citizens to influence healthcare is shaped by participatory spaces, and these spaces can potentially be reshaped by participants’ acts of citizenship. Our research highlights spatial aspects of participation that may contribute to exclusion from participatory initiatives, and future research should investigate this further.

We have shown that spatial dimensions of participation must be taken into account to understand whether and how official appeals to ‘active citizenship’ (Marinetto, 2003) are realised in practice. Grassroots acts of citizenship may open up unpredictable, but possibly more productive participatory ‘spaces for change’ (Cornwall and Coelho, 2006) and bring new modes of problem solving to healthcare improvement. Prioritising spatial governance (that is, creating and managing participatory space ‘from above’) over creating enabling environments for participation is likely to result in exclusionary systems. Sensitivity to the spatiality of participation and to the roles some citizens can play in creating spatial assemblages for collaboration will be crucial if participatory initiatives are to allow acts of citizenship – and the healthcare improvements that may result – to flourish.
